# BK Virus Encephalitis in HIV-Infected Patients: Case Report and Review

**DOI:** 10.1155/2017/4307468

**Published:** 2017-02-23

**Authors:** Luciana Antoniolli, Rafael Borges, Luciano Z. Goldani

**Affiliations:** Infectious Disease Section, Hospital de Clínicas de Porto Alegre, Universidade Federal do Rio Grande do Sul, Ramiro Barcelos 2350, 90640-000 Porto Alegre, RS, Brazil

## Abstract

Encephalitis and meningitis due to BKPyV are unusual and emerging condition. Only a few cases of BKPyV encephalitis have been reported in hematopoietic stem cell transplant recipients, with the majority of cases presenting with concurrent hemorrhagic cystitis and HIV-infected patients. The authors report two HIV-infected patients with the diagnosis of BKPyV encephalitis and discuss the main clinical, diagnostic, and therapeutic aspects of this infection in patients with AIDS. Physicians should be aware to recognize the main clinical features and diagnose BKPyV central nervous infection in the setting of AIDS.

## 1. Introduction

BK virus (BKPyV) JC virus (JCPyV) and simian 40 (SV40) are members of the Polyomaviridae family [1]. BKPYV infection affects 60–90% of the general population and occurs during childhood via the respiratory tract and is usually asymptomatic [2]. The virus remains latent after infection, particularly in the kidneys and other tissues including the brain [1, 2]. Viral reactivation occurring especially in immunocompromised patients may manifest as hemorrhagic cystitis, ureteral stenosis, tubulointerstitial nephritis, retinitis, encephalitis, and pneumonia [3]. Encephalitis and meningitis due to BKPyV are unusual and emerging condition with most cases being reported in HIV-infected patients or after transplantation [4, 5]. The authors report two HIV-infected patients with the diagnosis of BKPyV encephalitis and discuss the main clinical, diagnostic, and therapeutic aspects of this infection in patients with AIDS.

## 2. Case Reports

### 2.1. Case  1

A 52-year-old man was admitted to the emergency room because of a headache, fever (39°C), alternating with hypothermia, confusion, and weakness in the left leg. The patient also reported diarrhea, loss of appetite, and weight loss of 12 kilograms in the past three months. He had been diagnosed with HIV infection a few days before his admission. He denied previous diseases, using continuous medication, and previous hospital admissions or surgeries.

**Figure 1:**
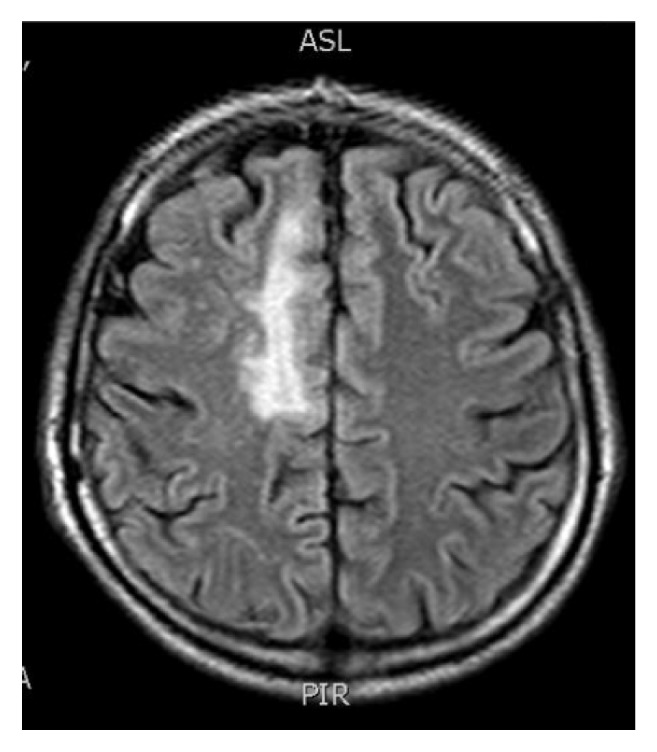
A magnetic resonance image showed a hyperintensity in T2 on the upper and lower right frontal gyri, in the left occipital lobe, and lesions with annular enhancement in the deep upper left temporal sulcus (case report 1).

At the physical examination, the patient presented a regular status with confusion, isochoric and photoreactive pupils, and no signs of meningeal irritation, but a loss of force in the left leg. Computed tomography (CT) scan showed an area of hypodensity in the white matter on the upper convexity of the right frontal lobe. A magnetic resonance image (MRI) showed a hypersignal in T2 on the upper and lower right frontal gyri, in the left occipital lobe, and lesions with ring enhancement in the deep upper left temporal sulcus (Figure 1). Laboratory exams showed a lymphocyte CD4 count of 94 cells/mm^3^, and HIV viral load of 479,365 copies ml. A reactive serological test for syphilis showed a titer 1 : 8, which was previously treated with penicillin G benzathine. A lumbar puncture was performed, and the cerebrospinal fluid (CSF) analysis showed 3 leucocytes/mm3, proteins of 28 mg/dL, and glucose of 39 mg/dL. CSF cultures were negative for bacteria, mycobacteria, and fungi. PCR testing for JCPyV virus was negative. However, PCR testing for BKPyV was positive. Detection of BKPyV consisted of a semi-nested PCR with two 20-base oligomer primers (PEP-1 and PEP-2) followed by second round PCR with 40-nucleotide sequence (BEP-1 and PEP-1), as previously described [6]. The length of the BKPyV targeted for amplification was 176 nucleotide pairs. At this same hospitalization, the patient was diagnosed with pulmonary tuberculosis, and a regime of rifampin, ethambutol, pyrazinamide, and isoniazid was provided. The patient was started on highly active antiretroviral therapy (HAART). However, he presented signs of sepsis of unknown origin twenty days after the admission and died three days later.

### 2.2. Case  2

**Figure 2:**
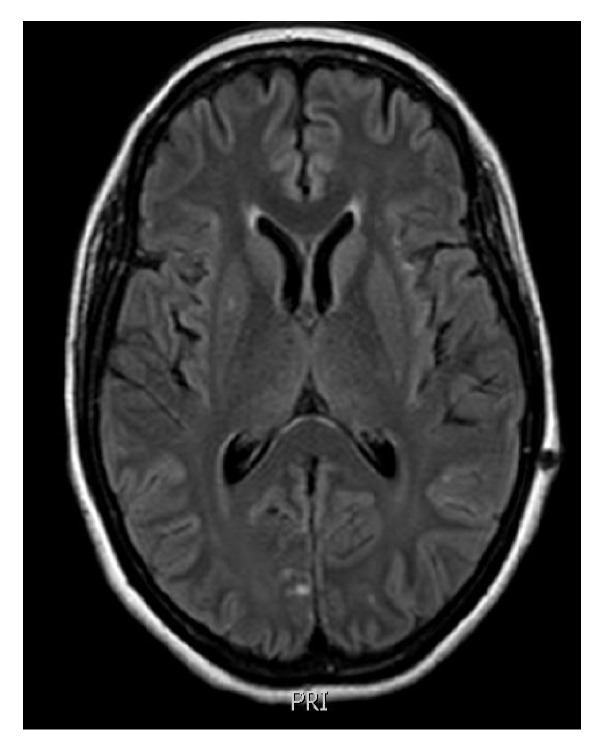
Cerebral MRI currently shows mild volume loss with scattered FLAIR hyperintensities and asymmetric lesions without mass effect (case report 2).

A 45-year-old woman was admitted to this hospital because of headaches, epigastric pain, nausea, vomiting, asthenia, dyspnea, loss of appetite, and fever. She had been diagnosed with HIV infection one month before. The patient was taking sulfadiazine and pyrimethamine due to a presumptive diagnosis of neurotoxoplasmosis in a previous recent hospitalization. On the physical examination, the patient was lethargic, with pain at palpation. She presented a severe immunosuppression with a CD4-T lymphocyte count of 23 cells/mm^3^ and a viral load of 167,580 copies/ml. Cerebral MRI showed mild volume loss with scattered FLAIR hyperintensities and asymmetric lesions without mass effect (Figure 2). The cerebrospinal fluid analysis showed 8 leucocytes/mm3, protein 133 mg/dL, and glucose 40 mg/dl. Cultures for bacteria, mycobacteria, and fungi were negative. PCR testing for herpes simplex virus types 1 and 2, herpes zoster virus, Epstein-Barr virus, and JC virus was negative. However, the sample was positive for the presence of BKPyV and cytomegalovirus. She continued to receive empiric treatment for neurotoxoplasmosis. During the hospitalization, she presented impaired renal function, which was attributed to sulfadiazine. It was also considered the hypothesis of BK renal infection, reinforced by a positive PCR testing for BKPYV in the urine. The patient was started on HAART (zidovudine, lamivudine, and efavirenz). She was discharged after 62 days of hospitalization with partial neurological improvement.

## 3. Discussion

BKPyV encephalitis has been reported in patients with depression of their immune function including patients with renal allograft: hematooncological diseases undergoing chemotherapy, bone marrow transplantation, long-term steroid therapy, and HIV [4–11]. However, BKPyV has been detected in asymptomatic apparently healthy and immunocompetent individuals, with self-limited clinical manifestations [12, 13]. To date, since the first report of BKPyV associated neurological infection almost 20 years ago, only eight cases have been described in patients with AIDS (Table 1) [14–22]. As observed in our two patients, the most common symptom is a headache. Other signs of neurological impairment include seizures, progressive mental deterioration, dysarthria, hallucinations, visual disturbances, and in one case paraplegia. BKPyV retinitis has been described in AIDS patients [20]. Imaging of CNS infection by BKPyV is characterized by a preferential involvement of the periventricular and pial surfaces of the brain parenchyma. In fact, magnetic resonance imaging of the cases of BKPyV meningoencephalitis has shown areas of increased signal intensity of the periventricular white matter of brain while the cortex is generally spared [5, 9]. Meningeal contrast enhancement along with increased meningeal thickness also occurs when infection spread to meningitis is present. Deep white matter of the cerebellum is generally spared while deep gray matter structures are compromised. As shown in Table 1, the diagnosis of BKPyV encephalitis has usually been established by PCR testing of the CSF in the majority of the patients and sometimes complemented or not by BKPyV PCR of the brain biopsy specimen. The lack of demonstration of the virus in the brain tissue sample has been frequently observed in the reported cases, either by immunohistochemistry or by in situ hybridization. If a patient shows neurological symptoms without a positive serology and PCR for other pathogens (namely, viral agents such as JCV, herpes simplex virus, and cytomegalovirus), while a positive PCR for BKPyV is present in the CSF/brain tissue, it strongly favors the diagnosis of a BKPyV meningoencephalitis.

There is no established treatment of BKPyV infection. Antiviral agents such as cidofovir, leflunomide, IVIG, and quinolones have been used in refractory cases of BKPyV nephropathy and cystitis [22–25]. However, the effectiveness of these agents is doubtful and some of them can cause severe side effects. However, in the few reported cases of BKPyV encephalitis, therapy has been restricted to the use of antiretroviral therapy. Consequently, overall mortality of the reported cases has been high, 70% of the reported cases in the literature. In summary, BKPyV meningoencephalitis is an emerging disease in HIV-infected patients. Physicians should be aware to recognize the main clinical features and diagnose BKPyV meningoencephalitis. Specific antiviral therapy is an urgent need for treatment of BKPyV infection.

## Figures and Tables

**Table 1:** Characteristics of HIV-infected patients with BKV encephalitis.

Reference age, sex	HIV parameters	Clinical presentation	Image findings	CSF parameters	Diagnosis	Other organs involved	Proposed treatment	Outcome
Current study 52, male	CD4: 94 cells/mm3 HIV VL: 479,365/copies mL	Fever, confusion, and weakness of the lower extremities	CT: hypodensity in the white matter on the upper convexity of the right frontal lobe MRI: hyperintensity in T2 on the upper and lower right frontal gyri, on the left occipital lobe, and lesions with ring enhancement in the deep upper left temporal sulcus	Opening pressure: 12.5 cm H_2_O Leuc: 3 Prot: 28 Gluc: 39 Bacterial, *M. tuberculosis *cultures, cryptococcal antigen, and JC PCR testing: negative	PCR testing positive for BKV in CSF	None	HAART	Death

Current study 45, female	CD4: 23 cells/mm3 HIV VL: 167,580/copies/mL	Fever, headache, epigastric pain, nausea, vomiting, and asthenia	CT: ring enhanced lesion without signs of activity MRI: a residual nodular lesion with ring enhancement	Leuc: 8 Prot: 133 Gluc: 40 Bacterial and *M. tuberculosis* cultures, *Cryptococcus* antigen, JC, and *M.tuberculosis* PCR testing: negative	PCR testing positive for BKV in CSF	Positive PCR for BKV in urine sample; kidney biopsy was not performed	HAART	Alive

Jørgensen et al. 29, male		Deafness and dizziness, renal dysfunction, and progressive retinitis	NA	NA	Positive PCR and IHC BKV from CSF and brain tissue, respectively	PCR and histochemical staining for BKV of PM samples from eye tissue, kidneys, and PBMCs	NA	Death

Lesprit et al. 44, male	CD4: 65 cells/mm3 Other diseases: large cell non-Hodgkin lymphoma	Hemorrhagic cystitis + progressive paraplegia	MRI: diffuse areas of increased signal intensity of the periventricular white matter	Negative for JC on PM biopsy	PCR testing positive for BKV from CSF and brain tissue	Positive PCR BKV from urine, blood, and bone marrow samples PM: positive PCR for BKV in the kidney, bladder, mesenteric node, and stomach tissue samples	NA	Death

Bratt et al. 26, male	NA	Dizziness and neurogenic deafness; progressive atypical bilateral peripheral retinitis 6 months after the onset of neurological symptoms	MRI: increased meningeal contrast enhancement and increased meningeal thickness	Leuc: 8 (2% neutrophils; 6% mononuclear)	PM biopsy of brain tissue	Immunohistochemical staining for BKV in PM biopsy of kidney	NA	Death

Vallbracht et al. 27, male	AIDS status was scored as C2 (CDC classification)	Headache; vomiting; disturbances of coordinative and mnemonic functions	CT: marked internal hydrocephalus with periventricular lucencies	Normal opening intracranial pressure Leuc: 15 Gluc: 58 Prot: 70	Positive BKV Southern blot in CSF	Positive PCR BKV in PM examination of kidneys, lungs, and central nervous system	NA	Death

Vidal et al. 43, male	CD4: 37 cells/mm3 Other diseases: cryptococcal meningitis; tuberculous meningitis	Headache, mental confusion, and dysarthria and ataxia	MRI: lesions in the gray matter of the left temporoparietal lobe and right occipital lobe; slight enhancement of the meninges	Leuc: 12 (79% lymphocytes, 10% monocytes) Prot: 146 Gluc: 46	PCR testing positive for BKV in CSF stereotactic biopsy of the brain: PCR positive for BKV	No	HAART	Alive

Hedquist et al. 29, male	NA	Several circular lesions in the retina; diminished visual acuity and deafness	NA	NA	PCR testing positive for BKV in the CSF	PCR for BKV and CMV in PM retina, choroid, vitreous, ciliary body, and sclera samples	NA	Death

Kinnaird and Anstead 48, male	NA	Ataxia, cognitive deficit, and dysarthria	MRI multifocal and infratentorial foci of hyperintensity	NA	NA	PCR positive for BKV; IHC antibodies positive for simian 40 in the urine	HAART	Alive

Garavelli and Boldorini 37, male	NA	Fever, headache, and altered mental status	NA	NA	PCR testing positive for BKV in the CSF	None	HAART	Alive

Bárcena-Panero et al. (case series of 13 patients) 26–56 years, male and female	HIV	Signs and symptoms related encephalopathy, meningitis	NA	NA	PCR testing positive for BKV in the CSF	None	NA	NA

HIV VL = HIV viral load; CT = computed tomography; MRI = magnetic resonance image; PM = post mortem; Leuc = leucocytes; cells/mm3; Prot = proteins; mg/dL; Glu = Glucose; mg/dL; HAART = highly active antiretroviral therapy; NA = not available; IHC = immunohistochemistry.
